# Validation of the Electronic Version of the International Index of Erectile Function (IIEF-5 and IIEF-15): A Crossover Study

**DOI:** 10.2196/13490

**Published:** 2019-07-02

**Authors:** Rob A A van Kollenburg, Daniel Martijn de Bruin, Hessel Wijkstra

**Affiliations:** 1 Department of Urology Amsterdam University Medical Centers, Location Amsterdam Medical Center University of Amsterdam Amsterdam Netherlands; 2 Signal Processing Systems Eindhoven University of Technology Eindhoven Netherlands

**Keywords:** ePROM, smartphone, surveys and questionnaires

## Abstract

**Background:**

Patient-reported outcome measures (PROMs) are increasingly used to measure patient’s perspective of functional well-being, disease burden, treatment effectiveness, and clinical decision making. Electronic versions are increasingly feasible because of smartphone and tablet usage. However, validation of these electronic PROMs (ePROMs) is warranted for justified implementation. The International Index of Erectile Function (IIEF) 5 and 15 are widely used PROMs in urology to measure erectile dysfunction. Measurement reliability and validity testing of the IIEF ePROMs are essential before clinical application.

**Objective:**

The aim of this study was to assess reliability and validity of an ePROM version of both IIEF-5 and 15.

**Methods:**

This study included 179 patients from our urology outpatient clinic. It also had a randomized crossover design—participants completed either a paper and electronic IIEF-5 or 15 or twice completed an electronic version—with a 5-day delay. Internal consistency was assessed using Cronbach alpha and Spearman-Brown coefficient, test-retest reliability using the intraclass correlation coefficient (ICC), and convergent validity using the Pearson and Spearman correlation coefficient.

**Results:**

A total of 122 participants completed the study. Internal consistency was excellent for the electronic IIEF-5 (ICC 0.902) and good to excellent for the domains of the IIEF-15 (ICC 0.962-0.834). Test-retest reliability was excellent for the IIEF-5 (ICC 0.924) and good to excellent for the domains of the IIEF-15 (ICC 0.950-0.778). Convergent validity was excellent for the IIEF-5 and IIEF-15, with a correlation of r=0.923 and r=0.951, respectively.

**Conclusions:**

We successfully introduced patient-acceptable ePROM versions of the IIEF-5 and IIEF-15. This study’s results demonstrate that the ePROM versions of the IIEF-5 and IIEF-15 can be reliably implemented, as outcomes are reliable and in accordance with findings of the paper version.

**Trial Registration:**

ClinicalTrials.gov NCT03222388; https://clinicaltrials.gov/ct2/show/NCT03222388

## Introduction

### Background

The International Index for Erectile Function (IIEF) is a patient-reported outcome measure (PROM), widely used in urology to measure erectile dysfunction (ED), applied both in clinical research and in daily clinical practice [1]. The 15-item version was developed by Rosen in 1997, and a 5-item short version followed in 1999 [2,3]. Translations into over 32 languages and validation of these translations followed [1,4,5]. Electronic PROMs (ePROMs), the electronic version of PROMs, are increasingly used, as the internet is easily accessible through mobile devices. The standard PROM is shifting from conventional paper and pen toward electronic administration, making ePROMs the (upcoming) new standard [6]. Attributing factors are smartphone use and subsequent development of patient-focused apps. Advantages of electronic administration are feasibility, automated calculations, reduced missing and ambiguous data, and increased compliance [7]. However, simple digitalization of existing PROMs does not assure reliability of ePROMs as administration, and subsequently outcomes, may be altered [6,8]. Therefore, reliability testing is advised to assure quality of ePROMs [8]. The extent of ePROM testing depends on the changes made during the PROM to ePROM transformation. Layout changes, for example, splitting the format into single questions, can be classified as a moderate level of modification [8]. For moderate-level modifications, a formal equivalence assessment of the electronic measure is advised, to show no significant difference in paper and electronic PROM scoring [8]. Given the fact that smartphone- and tablet-feasible ePROM versions of the IIEF-5 and 15 will probably include layout changes, reliability and validity testing of the IIEFs is therefore needed to assure outcome quality.

### Aim

The primary objective of this study was to develop an ePROM version of both the IIEF-5 and IIEF-15 and test reliability and validity in a male population.

## Methods

This observational study was conducted in a tertiary medical center, the Amsterdam University Medical Centers (UMCs), location Amsterdam Medical Center. The study received an ethics review waiver from the Institutional Review Board (W17.281), and the study was registered on Clinical Trial.gov (NCT03222388).

### Study Population

Male patients visiting the outpatient clinic of the urologic department were eligible for participation, patients were enrolled during a 6-month period, from July 2017 to December 2017. Screening for study eligibility (eg, inclusion and exclusion criteria and general health status) was based on information in the electronic patient file. Screening was performed by a medical doctor (RK, the primary author). Eligible patients were approached at the outpatient clinic before consultation. When interested, patients were informed about the study, and written informed consent was obtained. Inclusion criteria comprised males ≥40 years of age, in possession of an electronic device (smartphone/tablet/laptop), and fluent in Dutch. Exclusion criteria were adjustment of treatment during consultation (especially ED treatment), unable to provide informed consent, or unfit according to the medical doctor (eg, poor general health status).

### International Index for Erectile Function 5 and 15

The IIEF-15 comprises 15 items divided into 5 domains: erectile function, orgasmic function, sexual desire, intercourse satisfaction, and overall satisfaction, respectively. The IIEF-5 comprises 5 items from the IIEF-15, 4 from the erectile function domain, and 1 from intercourse satisfaction. Response options for each item ranged from 1 to 5, and occasionally the option “0,” depicting no sexual stimulation/intercourse. Scores are summed. Both versions have official Dutch translations [2,4].

### Study Design

A total of 179 participants were randomly assigned by the database management system (DMS) to the IIEF-5 or IIEF-15. Participants were hereafter randomly assigned to 2 groups: electronic version followed by electronic version (EE) or paper version and electronic version (PE). Primarily, participants in the PE group would randomly fill out either the paper or electronic IIEF to correct for order effects. This resulted in 6 different groups: (1) IIEF-5 paper electronic, (2) IIEF-5 electronic paper, (3) IIEF-5 electronic, (4) IIEF-15 paper electronic, (5) IIEF-15 electronic paper, and (6) IIEF-15 electronic. Participants were stratified on the basis of age <60 or ≥60, to improve group homogeneity on the basis of expected experience with internet/mobile devices.

### Study Methods

Participants assigned to a group with paper IIEF received this PROM in a sealed envelope during inclusion. The paper IIEF was returned to the researcher by an included return envelope with a stamp. Received paper IIEFs were coded and data were entered in the DMS. In case of missing data, the input was left blank. Participants received 2 emails containing a link to the ePROM, which could be completed at home at any convenient moment. The first invitation was sent 1 day postinclusion. A second invitation was sent 5 days after completion of the first ePROM. Reminders were sent twice, with a 3-day delay. If necessary, a personal reminder followed. The emails contained a link that redirected to a Web-based questionnaire. The first questionnaire started with several general questions, followed by either instructions for paper IIEF administration or the ePROM IIEF. This second questionnaire started with instruction or ePROM, followed by several evaluation questions.

### Electronic Patient-Reported Outcome Measure System

**Figure 1:**
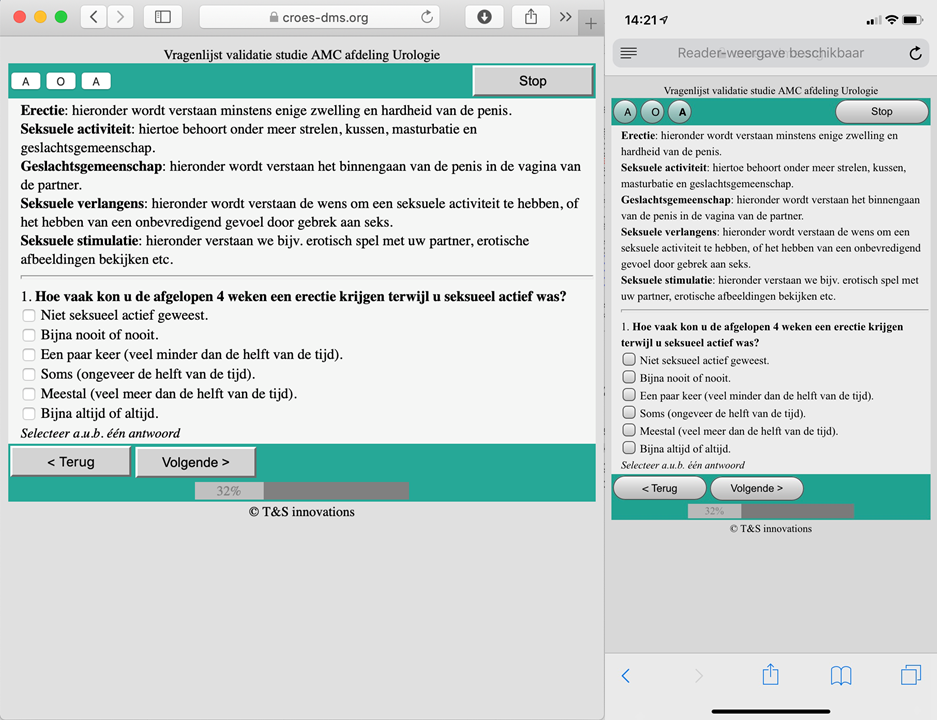
Screenshots of the ePROM displayed in a browser (Safari, left) and on a mobile device (iPhone, right).

The electronic questionnaire system for IIEF administration was built as part of the DMS (available for specific users at ts-innovations.com) [9]. The system was equipped with an ePROM module and automated invitations. The system worked as a Web-based environment with an identical interface across platforms (eg, Safari, Chrome, computer, and smartphone). The system displayed one PROM item at a time, and the patient had to click for the next question. This made it possible to display almost all information on the screen, without the need for scrolling. A system preview is presented in Figure 1.

### Electronic Patient-Reported Outcome Measure User Experience and Feasibility

After completion of the study, participants were asked about their willingness and preference to complete either only the PROM or ePROM or both. In addition, participants were asked to rate the overall ePROM quality on a scale of 1 to 10.

### Statistical Methods

Descriptive analyses were used for comparison of patient characteristics and feasibility outcomes. A 2-sided alpha level of .05 was considered statistically significant. Statistical analyses were performed using SPSS version 24.0 (SPSS inc).

### Sample Size

A total sample size of 172 participants was calculated for this study.

#### Sample Size: Paper Version and Electronic Version Groups

A 2-sided 95% CI was computed using the large sample normal approximation for an intraclass correlation on the basis of 2 PROMs, and it will extend about 0.100 from the observed intraclass correlation when the expected intraclass correlation is 0.800. This resulted in a sample size of 51. Anticipating a 20% dropout resulted in a sample size of 61 participants per PROM, thus resulting in 122 participants in total.

#### Sample Size: Electronic Version Followed by Electronic Version

A 2-sided 95% CI was computed using the large sample normal approximation for an intraclass correlation based on 2 PROMs, and it will extend about 0.100 from the observed intraclass correlation when the expected intraclass correlation is 0.880. This resulted in a sample size of 21. Anticipating a 20% dropout resulted in a sample size of 25 participants, thus resulting in 50 participants in total. The expected ICC of .88 was extracted from the Dutch IIEF-5 translation [4]. All sample sizes were calculated with the nQuery advisor software, provided by the Amsterdam UMC.

### Measurement Properties

The measurement properties were tested by the following methods:

1. The *internal consistency* is a measure of the extent to which items in a questionnaire scales and subscales are correlated, thus measuring the same concept [10]. The internal consistency was calculated for both paper and electronic IIEF data from the PE groups by Cronbach alpha or Spearman-Brown coefficient for 2-item subscales. An alpha ≥.9 reflected an excellent internal consistency, .9> alpha ≥ .8 reflected good consistency, and .8> alpha ≥.7 reflected acceptable internal consistency.
2. The *test-retest reliability* is the reliability of a test over time. The agreement between 2 repeated measurements was addressed with use of the ICC. These results were calculated based on the EE group results.
3. *Convergent validity* was also assessed. Support for this type of validity is provided if the total scale score and the subscale scores of the electronic version correlate substantially with the concerning scores of the original paper version. Convergent validity was analyzed using the Pearson correlation coefficient (*r*) or, when appropriate, Spearman rank correlation coefficient (rs) to determine the strength of the association between the paper and electronic IIEF.

For the ICC, a 2-way mixed-effect model, single measurement, and absolute agreement model was used. An ICC ≥0.9 reflected an excellent reliability, 0.9> ICC ≥0.75 reflected good reliability, and 0.75> ICC ≥0.5 reflected acceptable reliability, and <0.5 reflected poor reliability [11]. Pearson values *r* ≥0.5 reflected strong correlation, 0.5> *r*>0.3 reflected moderate correlation, and a 0.3> *r*>0.1 reflected weak correlation. A rank correlation of rs ≥0.5 reflected strong correlation, 0.5> rs >0.3 reflected moderate correlation, and 0.3> rs >0.1 reflected weak correlation [12].

### Data Safety

Data safety was guaranteed, as the emailed link redirected participants to a safe, validated, secured, Web-based environment. Information was directly stored in the DMS. No information was saved on the device itself, and all communication with the DMS was via an encrypted connection. The DMS was certified to store medical data (ISO9001, 14001, 27001:2013, and NEN7510). This was in line with Dutch guidelines and law concerning electronic collection of medical information.

## Results

### Participant Characteristics

**Figure 2:**
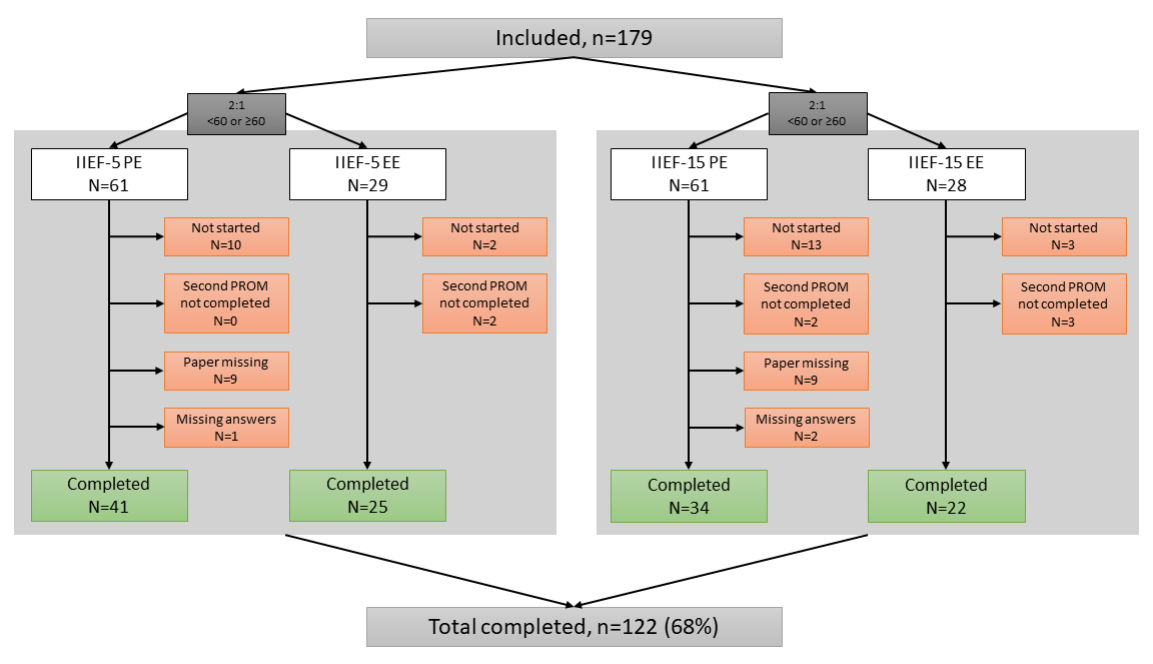
Participant inclusion and group allocation criteria (dark grey boxes). In the white boxes, the boxes contain the number of included participants per group. In the red boxes, the boxes contain excluded participants with reasons for exclusion. The green boxes show the number of included participants in the final analyses. EE: electronic version followed by electronic version; IIEF: International Index of Erectile Function; PE: paper version and electronic version; PROM: patient-reported outcome measure.

A total of 179 men were included in this study. A total of 122 participants completed the study and were included in the final analysis. Figure 2 provides an overview of participant allocation over groups, number of participants who completed the study, and numbers and reasons for participant exclusion. The overall mean age was 61.3±9.5 years (range 41-81 years). An extensive overview of participant characteristics is available in Multimedia Appendix 1. The mean time between (e)PROM completion was 7.26±4.23 days, ranging from 5 to 32.

### Internal Consistency

The internal consistency of the IIEF-5 is excellent for both the paper and electronic version (Table 1). The internal consistency for the paper IIEF-15 domains is good to excellent, ranging from 0.846 to 0.971.

**Table 1:** Internal consistency measured by Cronbach alpha or Spearman-Brown coefficient.

Measure		Paper	Electronic
IIEF^a^-5		.954^b^	.902^b^
**IIEF-15**		**.974^b^**	**.840^b^**
	Erectile function	.955^b^	.962^b^
	Orgasmic function	0.971^c^	0.937^c^
	Sexual desire	0.887^c^	0.848^c^
	Intercourse satisfaction	.935^b^	.917^b^
	Overall satisfaction	0.890^c^	0.924^c^

^a^IIEF: International Index for Erectile Function.

^b^For Cronbach alpha.

^c^For Spearman-Brown coefficient.

**Table 2:** Reliability of the electronic International Index for Erectile Function, calculated with the intraclass correlation coefficient.

Measure		Intraclass coefficient (95% CI)	*P* value
IIEF^a^-5 EE ^b^ (n=25)		0.924 (0.837-0.966)	<.001
**IIEF-15 EE (n=22)**			
	Erectile function	0.933 (0.847-0.971)	<.001
	Orgasmic function	0.778 (0.501-0.905)	<.001
	Sexual desire	0.823 (0.619-0.923)	<.001
	Intercourse satisfaction	0.950 (0.883-0.979)	<.001
	Overall satisfaction	0.878 (0.733-0.947)	<.001

^a^IIEF: International Index for Erectile Function**.**

^b^EE: electronic version followed by electronic version.

### Test-Retest Reliability Electronic International Index for Erectile Function

The test-retest reliability of the electronic version of the IIEF-5 was excellent with an ICC of 0.924 and 95% CI of 0.837-0.966 (Table 2). For the IIEF-15, the test-retest reliability was excellent for the domains erectile function and intercourse satisfaction, with an ICC of 0.933 and 0.950, respectively. The domains orgasmic function, sexual desire, and overall satisfaction were good with an ICC of 0.778, 0.823, and 0.878, respectively. All calculated correlation coefficients were significant (*P*<.001).

### Convergent Validity

The convergent validity for the IIEF-5 calculated by Pearson correlation coefficient was *r*=0.923 (Table 3). The overall correlation for the IIEF-15 scale was excellent, *r*=0.951. The correlations for the IIEF-15 subdomains ranged from 0.987 to 0.900. All calculated correlations were excellent and significant (*P*<.001).

**Table 3:** Concurrent validity across the paper and electronic International Index for Erectile Function, calculated with the Pearson correlation coefficient and Spearman rank correlation coefficient.

Measure		Correlation	*P* value
IIEF^a^-5 PE ^b^ (n=41)		0.923^c^	<.001
**IIEF-15 PE (n=34)**		**0.951^c^**	**<.001**
	Erectile function	0.987^c^	<.001
	Orgasmic function	0.947^d^	<.001
	Sexual desire	0.900^d^	<.001
	Intercourse satisfaction	0.973^c^	<.001
	Overall satisfaction	0.917^d^	<.001

^a^IIEF: International Index for Erectile Function.

^b^PE: paper version and electronic version.

^c^For Pearson correlation coefficient.

^d^For Spearman rank correlation coefficient.

**Table 4:** Feasibility outcomes.

Evaluating question	IIEF^a^-5	IIEF-15	*P* value
Willingness to complete either paper, electronic, or both IIEF	Only electronic 6 (15%); Only paper 6 (15%); Both 27 (69%)	Only electronic 8 (26%); Only paper 1 (3%); Both 22 (71%)	.81
Preference to complete either the paper or electronic IIEF	Electronic 25 (64%); Paper 8 (21%); None 6 (15%)	Electronic 18 (58%); Paper 8 (26%); None 5 (16%)	.52
Electronic IIEF: overall rating	7.8 (SD 1.3; range 4-10)	7.8 (SD 1.0; range 6-10)	—^b^

^a^IIEF: International Index of Erectile Function.

^b^Not applicable.

### Feasibility

Participants preferred an electronic version of the IIEF. After completion of both the PROM and ePROM IIEF, 69% of the IIEF-5 and 71% of the IIEF-15 participants were willing to complete both paper and electronic versions (Table 4). A vast majority preferred of the electronic versions with 64% and 58%, respectively. These numbers are similar to other studies [8]. Overall rating was 7.8 for both the IIEF-5 and IIEF-15.

### Participant Dropout

The actual number of participant dropout was higher than expected during sample size calculation. The actual number is 57 (32%), compared with the expected number of 28 (20%). A considerable number of participant dropout was a consequence of participants not starting at all (n=28, 49%) and paper IIEF’s not received by the authors (n=19, 33%). All reasons for dropout and missing data are shown in Figure 2.

## Discussion

### Principal Findings

The objective of this study was to develop ePROM versions of the IIEF-5 and 15 and test reliability and validity. The findings from this study demonstrated that both the electronic IIEF-5 and the IIEF-15 showed good-to-excellent internal consistency, test-retest reliability, and convergent validity to their paper version.

### Comparison With Literature

Outcomes of this study are in line with outcomes of previous validation studies of related PROMs. Reliability outcomes are in accordance with literature. The ICC of 0.924 for the IIEF-5 is in line with the ICC of 0.960 found in earlier research on electronic testing [13]. The ICC outcomes for the IIEF-15 ranging from 0.950-0.778 are in line with expectations of descriptive literature [8]. Findings are also in line with other review articles that compared ePROM validation outcomes [6,7]. It can be argued that the electronic IIEF-5 validation was redundant, as it was already shown on personal digital assistant (PDA) by Matthew et al [13]. However, a smartphone/computer differs from a PDA interface, and the study of Matthew at al used an interval of 30 min, whereas a washout period of at least 2 days is advised [8]. Therefore, we decided to include the IIEF-5 as well. Feasibility outcomes show that participants were willing to fill both versions, with a preference for the electronic version. This is in line with the increasing interest for ePROMs and their validation.

### Strengths and Limitations

The strengths of this study are the time between administrations, inclusion of the test-retest group, and administration at home. As other studies complied with a time delay of 30 min between administration moments, this study had a 5-day delay [6]. This reduced carryover effects, and this thus improved quality and reliability of the outcomes [8]. Furthermore, we decided to include a group that administrated the questionnaire twice electronically, hereby we could show the test-retest reliability of the electronic versions. A last strength of this study is the moment of administration. As invitations were sent via email, participants could complete the IIEF at home. This resulted in a standardized administration environment, which is identical to future administration factors; this improved the data quality. The limitations of this study concern the included population and dropout numbers. For this study, we chose the general population of our outpatient clinic. This resulted in a more heterogeneous population than specifically men with consultation for possible ED, the intended IIEF population. Men who are not sexually active were also included. We reasoned that this would not be a problem, as the objective was to show reliability and validity of the electronic IIEF version. Other issues that need to be addressed are the dropout numbers. The actual dropout number was higher (n=57, 32%) than anticipated (20%). All factors are shown in Figure 2. However, it is reasonable to assume that the missing data would not have significantly impacted the study outcome, as the obtained results were significant and in line with literature. The outcomes of this study are useful, as ePROMs are becoming more important in daily practice. For urology, it is likely that the ePROM version of the IIEFs will be used in clinical and research settings in the near future. Outcomes of this study are representable for IIEF application as ePROM as long as item presentation is in a similar, sequential manner.

### Conclusions

This study, with a randomized crossover design, demonstrated that the electronic IIEF-5 and IIEF-15 showed equivalence to the paper version. Electronic versions can therefore be used reliably in clinical and research settings. Outcomes are reliable and in accordance with findings of the paper version.

## Appendix

Multimedia Appendix 1 Complete overview of all participant characteristics.

Multimedia Appendix 2 All individual items of the paper-electronic and test-retest groups of the IIEF-5 and IIEF-15. IIEF: International Index of Erectile Function.

## Abbreviations

DMS: database management system
ED: erectile dysfunction
EE: electronic version followed by electronic version
ePROM: electronic patient-reported outcome measure
IIEF: International Index of Erectile Function
PDA: personal digital assistant
PE: paper version and electronic version
PROM: patient-reported outcome measure
UMC: University Medical Center
