# Supplementary material for: Validation of the Electronic Version of the International Index of Erectile Function (IIEF-5 and IIEF-15): A Crossover Study
Source: J Med Internet Res. 2019 Jul 2;21(7):e13490. doi: 10.2196/13490 (PMC6634948; doi:10.2196/13490)
Supplement: Multimedia Appendix 1 [file jmir_v21i6e13490_app1.docx]

Supplement 1:

Complete overview of all participants

|  | IIEF-5 P/E | IIEF-5 E/E | IIEF-15 P/E | IIEF-15 E/E | Total |
| --- | --- | --- | --- | --- | --- |
| Number included | 61 | 29 | 61 | 28 | 179 |
| Number completed study, included in analysis | 41 | 25 | 34 | 22 | 122 |
| Age | 59.6 (SD=8.9) (range 41-73) | 61.6 (SD=9.4) (range 41-76) | 62.3 (SD=10.5) (range 42-81) | 62.8 (SD=9.5) (range 44-77) | 61.3 (SD=9.5) (range 41-81) (p=0.843) |
| Education | Lower = 5  Middle = 19  High = 17 | Lower = 2  Middle = 9  High = 14 | Lower = 4  Middle = 11  High = 19 | Lower = 4  Middle = 10  High = 18 | Lower = 15 (12.3%)  Middle = 49 (40.2%)  High = 58 (47.5%) (p=0.685) |
| Native language | Dutch = 39  Other = 2 | Dutch = 24  English = 1 | Dutch = 33  English = 1 | Dutch = 21  Other = 1 | Dutch = 117 (95.9%)  English = 2 (1.6%)  Other = 3 (2.5%) (p= 0.536) |
| **Reason of consultation:** | | | | | |
| Voiding complaints | 6 (14.6%) | 4 (16%) | 7 (20.6%) | 1 (4.5%) | 18 (14.8%) (p=0.379) |
| Urolithiasis | 4 (9.8%) | 6 (24.0%) | 5 (14.7%) | 2 (9.1%) | 17 (13.9%) (p=0.368) |
| Carcinoma/FU | 25 (61.0%) | 11 (44.0%) | 18 (52.9%) | 16 (72.7%) | 70 (57.4%) (p=0.218) |
| Andrological | 4 (9.8%) | 1 (4.0%) | 2 (5.9%) | 0 (0.0%) | 7 (5.7%) (p=0.440) |
| Other | 2 (4.9%) | 3 (12.0%) | 2 (5.9%) | 3 (13.6%) | 10 (8.2%) (p=0.534) |
| **Medication:** | | | | | |
| BPH | 2 (4.9%) | 2 (8.0%) | 2 (5.9%) | 1 (4.5%) | 7 (5.7%) (p=0.961) |
| LUTS | 4 (9.8%) | 2 (8.0%) | 5 (14.7%) | 1 (4.5%) | 12 (9.8%) (p=0.637) |
| ED | 0 (0.0%) | 1 (4.0%) | 0 (0.0%) | 0 (0.0%) | 1 (0.8%) (p=0.271) |
| (anti)hormones | 5 (12.2%) | 1 (4.0%) | 2 (5.9%) | 1 (4.5%) | 9 (7.4%) (p=0.324) |
| (anti)hormones + BPH | 1 (2.4%) | 0 (0.0%) | 0 (0.0%) | 0 (0.0%) | 1 (0.8%) (p=0.961) |
| NA | 29 (70.7%) | 19 (76.0%) | 25 (73.5%) | 19 (86.4%) | 92 (75.4%) (p=0.577) |
