# Supplementary material for: Validation of the Electronic Version of the International Index of Erectile Function (IIEF-5 and IIEF-15): A Crossover Study
Source: J Med Internet Res. 2019 Jul 2;21(7):e13490. doi: 10.2196/13490 (PMC6634948; doi:10.2196/13490)
Supplement: Multimedia Appendix 2 [file jmir_v21i7e13490_app2.pdf]

Supplement 2:

All individual items of the paper-electronic and test-retest groups of the IIEF-5 and IIEF-15

IIEF – item	(Mean ± SD)	(Mean ± SD)
**PE groups**	**Paper**	**Electronic**
IIEF5 – 1	3.61 ± 1.28	3.63 ± 1.02
IIEF5 – 2	3.93 ± 1.60	2.02 ± 1.53
IIEF5 – 3	3.61 ± 1.87	1.78 ± 1.53
IIEF5 – 4	3.93 ± 1.77	2.49 ± 1.87
IIEF5 – 5	3.66 ± 1.78	1.63 ± 1.32
IIEF15 – 1	3.38 ± 1.91	2.00 ± 1.71
IIEF15 – 2	2.79 ± 2.17	2.00 ± 1.79
IIEF15 – 3	2.56 ± 2.34	1.18 ± 1.47
IIEF15 – 4	2.50 ± 2.40	1.18 ± 1.47
IIEF15 – 5	2.44 ± 2.26	1.21 ± 1.50
IIEF15 – 6	1.41 ± 1.52	2.24 ± 2.10
IIEF15 – 7	2.65 ± 2.39	1.21 ± 1.49
IIEF15 – 8	2.29 ± 2.10	1.35 ± 1.39
IIEF15 – 9	3.18 ± 2.04	1.82 ± 1.51
IIEF15 – 10	3.29 ± 2.02	2.15 ± 1.78
IIEF15 – 11	3.09 ± 1.38	3.06 ± 1.25
IIEF15 – 12	3.06 ± 1.10	3.00 ± 0.99
IIEF15 – 13	3.50 ± 1.29	3.47 ± 1.13
IIEF15 – 14	3.50 ± 1.31	3.50 ± 1.21
IIEF15 – 15	3.35 ± 1.30	3.38 ± 1.21
**EE groups**	**Electronic 1st**	**Electronic 2nd**
IIEF5 – 1	3.28 ± 1.31	3.28 ± 1.17
IIEF5 – 2	1.92 ± 1.58	1.76 ± 1.56
IIEF5 – 3	1.64 ± 1.66	1.96 ± 174
IIEF5 – 4	2.52 ± 2.00	2.80 ± 2.00
IIEF5 – 5	1.92 ± 1.61	1.64 ± 1.38
IIEF15 – 1	1.55 ± 1.22	1.50 ± 1.41
IIEF15 – 2	2.05 ± 1.70	2.05 ± 1.86
IIEF15 – 3	1.64 ± 1.59	1.59 ± 1.71
IIEF15 – 4	1.82 ± 1.87	1.27 ± 1.55
IIEF15 – 5	1.36 ± 1.47	1.27 ± 1.55
IIEF15 – 6	2.14 ± 2.08	2.36 ± 2.11
IIEF15 – 7	1.32 ± 1.43	1.27 ± 1.55
IIEF15 – 8	1.23 ± 1.27	1.18 ± 1.33
IIEF15 – 9	2.09 ± 1.77	2.00 ± 1.80
IIEF15 – 10	1.77 ± 1.34	1.68 ± 1.62
IIEF15 – 11	3.45 ± 1.22	3.27 ± 1.28
IIEF15 – 12	3.05 ± 1.09	3.18 ± 1.05
IIEF15 – 13	3.41 ± 1.14	3.45 ± 1.10
IIEF15 – 14	3.36 ± 1.40	3.59 ± 1.26
IIEF15 – 15	3.18 ± 1.14	3.09 ± 1.51
